# Effects and Adaptation of Visual-Motor Illusion Using Different Visual Stimuli on Improving Ankle Joint Paralysis of Stroke Survivors—A Randomized Crossover Controlled Trial

**DOI:** 10.3390/brainsci12091249

**Published:** 2022-09-15

**Authors:** Junpei Tanabe, Kazu Amimoto, Katsuya Sakai, Shinpei Osaki, Nao Yoshihiro, Tokuei Kataoka

**Affiliations:** 1 Department Physical Therapy, Hiroshima Cosmopolitan University, Hiroshima 731-3166, Japan; 2 Department Physical Therapy, Tokyo Metropolitan University, Tokyo 116-8551, Japan; 3 Department of Physical Therapy, Chiba Prefectural University of Health Sciences, Chiba 260-0801, Japan; 4 Department of Rehabilitation, Kansai Electric Power Hospital, Osaka 553-0003, Japan; 5 Department of Occupational Therapy, Kansai University of Health Sciences, Osaka 590-0482, Japan; 6 Department of Rehabilitation, Kurashiki Rehabilitation Hospital, Okayama 710-0834, Japan

**Keywords:** visual-motor illusion, sense of agency, maximum effort, ankle dorsiflexion function, cognitive flexibility, muscle tone, stroke patients

## Abstract

Visual-motor illusion (VMI) is an intervention to induce kinesthetic sensation from visual stimuli. We aimed to compare the effects of VMI of different visual stimuli on the paralyzed side ankle joint of stroke hemiplegic patients (hemiplegic patients) and to clarify their indication. We applied two types of VMI images of ankle dorsiflexion: ankle dorsiflexion without resistance (standard VMI (S-VMI)) and maximum effort dorsiflexion with resistance (power VMI (P-VMI)). Twenty-two hemiplegic patients were divided into two groups: Group A, which received S-VMI first and P-VMI one week later (*n* = 11), and Group B, which received P-VMI first and S-VMI one week later (*n* = 11). Immediate effects were evaluated. Outcomes were the dorsiflexion angle and angular velocity, degree of sense of agency (SoA), and sense of ownership. Patient’s characteristics of cognitive flexibility were assessed using the Trail making test-B (TMT-B). Fugl-Meyer assessment and the Composite-Spasticity-Scale were also assessed. P-VMI was significantly higher than S-VMI in SoA and dorsiflexion angular velocity. Additionally, the degree of improvement in dorsiflexion function with P-VMI was related to TMT-B and degree of muscle tone. Therefore, P-VMI improves ankle function in hemiplegic patients more than S-VMI but should be performed with cognitive flexibility and degree of muscle tone in mind.

## 1. Introduction

Hemiplegic stroke patients (hemiplegic patients) have abnormal balance function due to spasticity, muscle weakness, sensory loss, and motor dysfunction [1,2,3]. Previous studies have reported that hemiplegic patients with impaired ankle joint control due to spasticity of the triceps muscle on the paralyzed side, as well as dysfunction of the tibialis anterior muscle, have reduced balance ability, leading to decreased walking ability and an increased risk of falls [3,4,5]. Ankle joint movement is important for controlling the position of the body’s support base and for maintaining balance [6]. In addition, maintaining balance depends on the ankle joint’s range of motion and on good control of the ankle dorsiflexor and plantar flexor contraction [3,7]. Therefore, reducing ankle plantar flexor spasticity and improving ankle dorsiflexion function are important rehabilitation goals in stroke patients because they contribute to improving balance and walking ability.

Recently, visual-motor illusion (VMI), in which kinesthetic sensation is induced by visual stimulation, has been reported to be effective in improving motor function in hemiplegic patients [8,9,10,11,12,13,14]. VMI induces the illusion of movement in the subject’s limbs, even though they are not moving, by overlaying an image showing the movements of the limbs on the actual subject’s limbs for observation [15,16,17,18,19,20]. VMI has been reported to increase corticospinal tract excitability, elicit motor imagery, and activate brain regions associated with a sense of embodiment (sense of agency (SoA) and sense of ownership (SoO)) [8,17,21,22]. Furthermore, SoA elicited by VMI is associated with prefrontal cortex activity [22].

Mirror therapy (MT) has been reported to be effective as an intervention that uses visual stimuli to promote illusions, but it has been noted to promote interhemispheric inhibition because the non-paralytic limb is moved during MT [8]. On the other hand, VMI does not require movement of the non-paralytic side, making it easier to focus attention on the intervention limb. Furthermore, action observation therapy (AOT), which elicits motor imagery from visual stimuli, has also been reported to improve motor function in patients [23]. However, the degree of SoO and the degree of brain network activation are reported to be greater with VMI than with AOT [21]. Numerous previous reports have indicated that VMI led to improved upper limb function in hemiplegic patients [8,9,10,11,12]. In addition, for paralyzed ankle joints, VMI has led to an increase in the voluntary ankle dorsiflexion angle, suppression of muscle tone in the antagonist muscle, and improvement in standing movements and walking ability [13,14].

VMI has been reported to induce motor imagery similar to the joint motion [17] and to enhance motor imagery ability [24]. Therefore, it is possible that motor imagery may be induced depending on the strength of joint motion in the presented video. Furthermore, it has been reported that during motor imagery, the excitability of corticospinal tracts changes in relation to the intensity of the imagined muscle contraction [25]. SoA is related to the excitability of corticospinal tracts [26], and a sense of effort has been reported to enhance SoA [27]. Therefore, VMI with increased intensity of joint movements in the presented video (Power-VMI; P-VMI) may be more effective in improving motor function than Standard-VMI (S-VMI), which has been used to date. However, the images presented in previous VMI for the paralyzed ankle joint only show repeated ankle dorsiflexion [13,14], and no studies to date have considered the impact of the strength of joint motion.

One of the task characteristics of VMI is the need to maintain the motor imagery induced by the video image until the actual execution of the exercise. Retention of this motor imagery is mediated by cognitive flexibility function, which is closely related to the ability to execute the intended motor activity [28]. Kawasaki et al. [29] showed that the degree of improvement in motor function with AOT was related to cognitive flexibility function. We hypothesized that the degree of improvement in motor function and cognitive flexibility would be related to the degree of improvement in motor function with VMI. VMI elicits motor imagery by creating an illusion from visual stimuli, similar to the AOT task. Sakai et al. [30] also reported that motor imagery ability was related to lower limb function in patients with hemiplegia. Thus, the degree of improvement in motor function may be related to lower-extremity function with VMI. However, the effects of changed intensity in VMI on improving lower-extremity function in hemiplegic stroke patients and the relation thereof to cognitive flexibility have not yet been investigated.

Therefore, this study aimed to compare the effects of P-VMI and S-VMI on ankle dorsiflexion function in hemiplegic patients. Second, we investigated the relationship between the degree of improvement in ankle function with VMI and embodiment sensation, cognitive flexibility, and physical function to examine the indications for the two types of VMI.

## 2. Materials and Methods

### 2.1. Study Design

This study was an assessor-blinded randomized crossover controlled trial conducted according to the CONSORT checklist [31]. The participants were randomly assigned to Group A or Group B in equal numbers using a permuted block randomization method by hospital staff not involved in the study. Randomization codes were generated using a computerized random number-generator program. Participants drew a randomization code hidden inside a sealed opaque envelope. After the assignment, the allocator reported the group assignment to the study coordinator. Investigators evaluating the results of the interventions were unaware of the group allocation. Participants received both S-VMI and P-VMI with a 1-week interval between the two interventions. To examine the immediate effects, the outcomes were assessed directly before and after each intervention. Group A received S-VMI first and P-VMI 1 week later, while Group B received P-VMI first and S-VMI 1 week later.

This study was approved by the Ethics Committee of Kurashiki Rehabilitation Hospital (approval number: 1905). This study was registered in the University Hospital Medical Information Network Clinical Trials Registry (UMIN CTR number: UMIN000042431). Prior to participation, written informed consent was obtained from all participants in accordance with the Declaration of Helsinki.

### 2.2. Participants

The participants were hemiplegic patients (infarction or hemorrhage) who underwent inpatient rehabilitation at a Japanese hospital from December 2020 to March 2022. The inclusion criteria were: (1) first-episode hemiplegic patients, (2) who had no orthopedic disease, (3) in whom stroke impairment assessment of the distal lower extremity indicated a score of 1 or higher (slight dorsiflexion movement but forefoot not off the floor), (4) in whom the muscle tone of the triceps muscle on the paralyzed side of the leg showed a score of 1 or higher on the Modified Ashworth Scale [32], and (5) who had no higher brain dysfunction (unilateral spatial neglect, aphasia). Exclusion criteria were as follows: (1) a Mini-Mental State Examination score < 21 points, (2) presence of visual impairment, and (3) the inability to follow verbal instructions.

### 2.3. VMI Intervention

**Figure 1:**
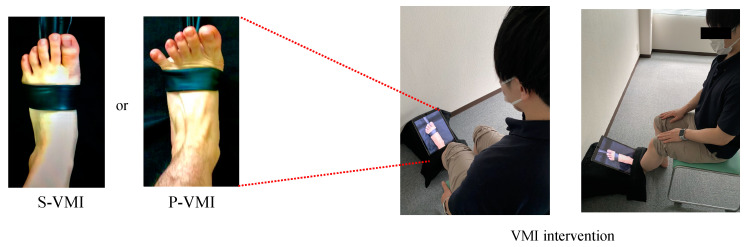
VMI intervention. (1) TheraBand: A TheraBand was used to apply resistance to the ankle dorsiflexion movement. (2) S-VMI: A TheraBand was wrapped around the foot, but no tension was applied. (3) P-VMI: Resistance was applied to the foot using the TheraBand to perform dorsiflexion with maximum effort. (4) Tablet computer: Videos of the ankle joint dorsiflexion movements of either S-VMI or P-VMI were projected on a tablet computer. (5) VMI intervention: Participants observed the video in a sitting position for 5 min. In P-VMI, strong contraction of the tibialis anterior muscle, extension movement of the toes, and activity of the extensor hallucis longus muscle tendon and extensor digitorum longus muscle tendon were clearly observed compared to S-VMI. Abbreviations: P-VMI, Power-VMI; S-VMI, Standard-VMI; VMI, visual-motor illusion.

The VMI videos included ankle dorsiflexion exercises under two conditions. Both videos were created using TheraBand (Thera-Band, Abilities, Tokyo, Japan). The S-VMI video showed ankle dorsiflexion movement with TheraBand wrapped around the foot, and no tension was applied. In contrast, in the P-VMI video, resistance was applied to the foot via TheraBand, and dorsiflexion was performed with maximum effort. Both the S-VMI and P-VMI videos recorded ankle joint movements on the non-paralyzed side. The number of ankle dorsiflexion movements was set to 60 per minute for both conditions [13,14], and video images were recorded using a tablet device (iPad Pro, Apple, Cupertino, CA, USA). The VMI video image was then inverted using video inversion software so that it appeared as dorsiflexion motion on the paralyzed side. During VMI, the patient was seated. The monitor was then presented over the paralyzed ankle joint and set up to maintain continuity between the actual and virtual lower extremities in the video (Figure 1). The examiner gave the patient the following instructions: “You do not have to actually move while observing the video; just imagine that you are moving your own ankle in the same way as the ankle movement in the video” [14].

### 2.4. Outcome Measures

The primary outcomes were the ankle dorsiflexion angle and ankle dorsiflexion angular velocity on the paralyzed side pre and post VMI training. The secondary outcome was the degree of SoA and SoO during the VMI. The cognitive flexibility of patients was assessed using the Trail Making Test B (TMT-B). In addition, the Fugl-Meyer assessment (FMA) lower extremity items, Stroke Impairment Assessment Set (SIAS) sensory items, and Composite-Spasticity Scale (CSS) were used to assess muscle tone.

#### 2.4.1. Assessment of Ankle Joint Dorsiflexion Movement

A digital video camera (EX-FC150; Casio, Tokyo, Japan) was used to collect ankle joint kinematic data. The camera was placed on the paralyzed side of the subject, and ankle joint dorsiflexion movements were recorded in the sagittal plane. The sampling frequency was 120 Hz. Three markers were placed on the lateral femoral epicondyle, lateral malleolus, and fifth metatarsal head on the paralyzed side [14].

To test ankle joint dorsiflexion movements, the participants sat on a height-adjustable bed so that their feet did not touch the floor. The participants were instructed to dorsiflex the ankle joint to a maximum [14]. The recorded video was analyzed using a two-dimensional (2D) motion analysis system (ToMoCo-Lite, Tosoh System; Saitama, Japan) to calculate joint angles and joint motion times [33]. For the within-tester reliability of the angle calculation using ToMoCo-Lite, the intraclass correlation coefficient ranged from 0.80 to 0.97 [34]. The ankle joint angle was defined as the line connecting the lateral femoral epicondyle to the lateral malleolus, and the line connecting the lateral malleolus to the fifth metatarsal bone [14]. The mean angle and standard deviation were calculated from 10 unchanged frames before and after the ankle dorsiflexion movement, using a 2D motion analysis system [35]. The start and end points of the joint motion were defined as the average angle of 10 frames without angle change before and after the ankle dorsiflexion movement, plus twice the standard deviation [35]. The ankle dorsiflexion time was calculated as the number of frames required to complete dorsiflexion divided by 120 (sampling frequency) using a 2D motion analysis system [14]. Ankle dorsiflexion angular velocity was calculated by dividing the ankle dorsiflexion angle by dorsiflexion time [14]. To measure the degree of improvement by VMI, the pre value was subtracted from the post value, and the amount of change was calculated. The ankle dorsiflexion angle and angular velocity values were averaged over five evaluations [36].

#### 2.4.2. Measuring SoA and Sense of SoO

The SoA and SoO that occurred during the VMI intervention were based on participant self-reports using a visual analog scale (VAS: 0 mm (no SoA or SoO) to 100 mm (SoA or SoO occurred)). For SoA, participants were asked, “How well did you feel you were able to control your ankle joint movement?” [37]. For SoO, participants were asked, “How much did the ankle joint in the image feel like part of your body?” [19].

#### 2.4.3. TMT-B

TMT-B involved interspersed numbers 1–13 and letters A–L, and the participants were asked to connect the numbers and letters of the Japanese alphabet alternately. The time from the start of the signal to the final point of the connection was measured using a digital stopwatch. No practice trials were provided for this test, and the test commenced once the task had been explained [29]. Arbuthnott and Frank [38] found a significant association between the TMT-B completion time and the ability to process multiple tasks. This suggests that the central executive system, which is at the core of cognitive flexibility, may represent the ability of the central executive system.

#### 2.4.4. FMA Lower Extremity Items

The FMA-lower extremity (FMA-LE), consisting of 17 items, was used to examine motor function and coordination in the affected lower extremity [39]. Total FMA-LE scores ranged from 0 to 34, with higher scores indicating a lower level of impairment.

#### 2.4.5. SIAS Sensory Items

The lower extremity sensory items of the SIAS are light touch (tactile) and position sense (position) [40]. Tactile and position sense scores ranged from 0 to 3, with lower scores indicating more severe impairment.

#### 2.4.6. Measurement of Ankle Plantar Flexor Muscle Tone

Ankle plantar flexor muscle tone was assessed using CSS, which consists of the degree of Achilles tendon reflex, resistance to passive full extension of ankle dorsiflexion, and degree of foot clonus. Scores ranged from 0 to 16, with scores of 0–9, 10–12, and 13–16 indicating mild, moderate, and severe spasticity, respectively [41].

### 2.5. Statistical Analysis

The normality of data distribution of all variables was tested using the Shapiro–Wilk test. The independent *t*-test, Mann–Whitney U test, and chi-square test were used to compare patient characteristics between the two groups. Time effects (pre- vs. post-intervention) and comparisons between conditions at each evaluation period (P-VMI vs. S-VMI) were analyzed using the paired *t*-test. G* power 3.1.9.2 (Heinrich Heine University, Dusseldorf, Germany)was used to calculate effect sizes before and after VMI from the mean, standard deviation, and correlation coefficient between the two groups [42]. Cohen’s d was calculated, and d < 0.4, d = 0.4–0.8, and d > 0.8 were defined as small, medium, and large effect sizes, respectively [43]. Rates of change {(Post value − Pre value)/Pre value} were calculated and compared using paired *t*-tests to identify differences in intervention methods (P-VMI and S-VMI). The degrees of SoA and SoO of P-VMI and S-VMI were compared using a paired *t*-test. The effect was determined according to the minimal clinically important difference (MCID) to evaluate whether the change in VMI was clinically significant. The change (from before to after the intervention) was considered clinically significant if it exceeded the MCID, which was defined as half the standard deviation (0.5 SD) of the pre-intervention [44,45,46]. Pearson’s product-moment correlation coefficient and Spearman’s rank correlation coefficient were used to investigate the relationship between the degree of improvement in the paralyzed side ankle joint dorsiflexion function (ankle joint dorsiflexion and angular velocity) and VMI: (1) degree of SoO; (2) degree of SoA; (3) TMT-B, an assessment of cognitive flexibility; and (4) physical function (CSS as an assessment of motor function, sensation, and muscle tone in the lower extremity). Statistical analysis was performed using SPSS 20.0 (SPSS Inc., Chicago, IL, USA), and statistical significance was set at *p* < 0.05.

#### Sample Size

For the sample size estimation, for each additional subject, the post hoc test of G* power 3.1.9.2 (Düsseldorf University; Düsseldorf, Germany) was used to estimate the Cohen’s d of the primary outcome. In addition, the power of the primary outcome was calculated, and subject recruitment was stopped when the power (1-β) exceeded 0.95 [24,47]. The results of the interim analysis showed that the effect sizes of ankle dorsiflexion angle and ankle dorsiflexion angular velocity before and after the P-VMI intervention were high: power for the ankle dorsiflexion angle was 0.97 and for the ankle dorsiflexion angular velocity was 0.99 (Table 1). Based on these results, we decided to discontinue the study enrollment after recruiting 22 participants.

## 3. Results

### 3.1. Participants

**Figure 2:**
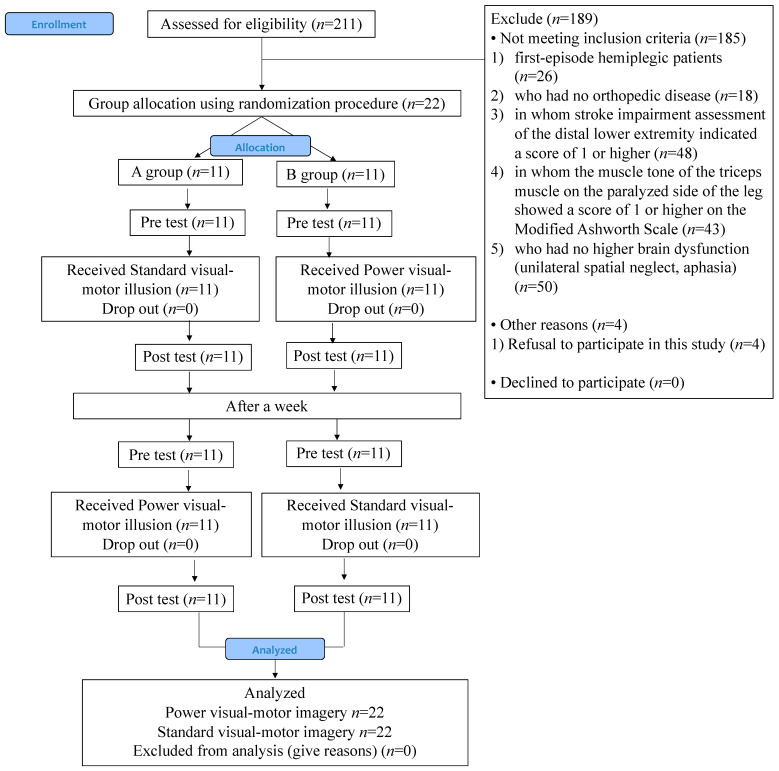
Flow diagram used for the selection of participants for this study.

The flowchart of the study is presented in Figure 2. Subjects were screened from 331 patients recruited between December 2020 and March 2022; 22 patients were enrolled and randomly assigned to either Group A (*n* = 11) or Group B (*n* = 11). All participants were eligible for the intervention, and no adverse events occurred during the study. Eleven participants in groups A and B performed both P-VMI and S-VMI. The sample size for P-VMI and S-VMI was 22 because the order of P-VMI and S-VMI was altered in groups A and B (Figure 2). The characteristics of the two groups are shown in Table 2. There were no statistically significant differences in the characteristics between groups A and B (Table 2).

To calculate the mean value of MAS scores, score 1+ was transformed to 2, and scores 2, 3, and 4 were transformed to 3, 4, and 5.

### 3.2. Comparison of Ankle Dorsiflexion Function between P-VMI and S-VMI

**Figure 3:**
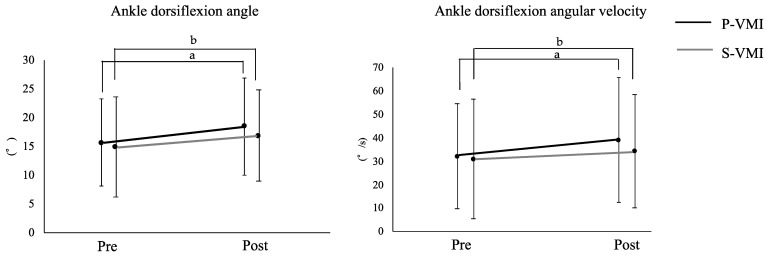
Results of Effect sizes pre and post VMI. a: Effect size of post hoc test is large. b: Effect size of post hoc test is medium. Abbreviations: P-VMI, Power-VMI; S-VMI, Standard-VMI; VMI, visual-motor illusion.

Table 3 shows the changes in the ankle dorsiflexion function pre and post intervention. On the paralyzed side, ankle dorsiflexion angle and angular velocity showed no significant differences between the pre values of P-VMI and S-VMI (dorsiflexion angle: t(21) = 1.388, *p* = 0.180; dorsiflexion angular velocity: t(21) = 0.741, *p* = 0.467). However, on the paralyzed side, ankle dorsiflexion angle post values were significantly increased as compared to the pre values for both P-VMI and S-VMI (P-VMI: t(21) = −4.309 *p* < 0.001; S-VMI: t(21) = −3.255, *p* = 0.004). The effect sizes were medium for S-VMI (d = 0.67) and large for P-VMI (d = 0.96; Figure 3). Post values of dorsiflexion angular velocity of the paralyzed ankle joint were significantly higher than the pre values for both P-VMI and S-VMI (P-VMI: t(21) = −4.199, *p* < 0.001; S-VMI: t(21) = −3.106, *p* = 0.005). The effect sizes were medium (d = 0.46) for S-VMI and large (d = 0.86) for P-VMI (Figure 3). Comparison of the post values showed that P-VMI was significantly higher than S-VMI (t(21) = 2.510, *p* = 0.020). However, comparison between interventions (P-VMI and S-VMI) using rate of change did not show significant differences for the ankle dorsiflexion angle and angular velocity (dorsiflexion angle: t(21) = −0.493, *p* = 0.627; dorsiflexion angular velocity: t(21) = 0.068, *p* = 0.946).

### 3.3. MCID

In the MCID(0.5SD) of the ankle dorsiflexion angle, P-VMI was 3.8 and S-VMI was 4.3, and of the ankle dorsiflexion angular velocity, P-VMI was 11.3 and S-VMI was 11.3. The amounts of changes in ankle dorsiflexion angle were 2.7 for P-VMI and 2.0 for S-VMI, and those in ankle dorsiflexion angular velocity were 7.0 for P-VMI and 3.3 for S-VMI, which were lower than the MCID values.

### 3.4. Relationship between the Degree of Improvement of Ankle Joint Function by P-VMI and S-VMI and the Sense of Embodiment (SoA and SoO)

Correlation analysis showed that the changes in dorsiflexion angle and dorsiflexion angular velocity of P-VMI were positively correlated with SoA (dorsiflexion angle and SoA: r = 0.518, *p* = 0.014; dorsiflexion angular velocity and SoA: r = 0.449, *p* = 0.036; Table 4). The change in the dorsiflexion angle of S-VMI was positively correlated with SoA (r = 0.493, *p* = 0.020). In the P-VMI and S-VMI, SoO was not significantly correlated with improved ankle function.

### 3.5. Relationship between Cognitive Flexibility and Degree of Improvement in Ankle Joint Function by P-VMI and S-VMI

Correlation analysis showed that the changes in dorsiflexion angle and dorsiflexion angular velocity with P-VMI were negatively correlated with TMT-B (dorsiflexion angle and TMT-B: r = −0.570, *p* = 0.006; dorsiflexion angular velocity and TMT-B: r = −0.449, *p* = 0.036; Table 4). No significant correlation was found between the dorsiflexion functions of S-VMI and TMT-B.

### 3.6. Relationship between Degree of Improvement in Ankle Function and Physical Function by P-VMI and S-VMI

There was a significant negative correlation between the change in dorsiflexion angular velocity of P-VMI and CSS, reflecting muscle tone assessment results (r = −0.430, *p* = 0.047; Table 4). No significant correlation was found between the degree of improvement in ankle dorsiflexion function and other physical functions in P-VMI and S-VMI.

### 3.7. Comparison of P-VMI and S-VMI for Sense of Embodiment

P-VMI was significantly higher than S-VMI, with P-VMI of 73.9 ± 11.3 and S-VMI of 62.0 ± 13.1 (t(21) = 3.862, *p* < 0.001) for SoA and P-VMI of 63.9 ± 10.5 and S-VMI of 59.2 ± 9.1 (t(21) = 2.547, *p* = 0.019) for SoO.

## 4. Discussion

This study compared images of two different types of visual stimuli, which has not been reported previously, and showed that these approaches resulted in different improvements in ankle joint function. Furthermore, this study investigated the relationship of the degree of improvement in ankle function, achieved with VMI, with embodiment sensation, cognitive flexibility, and motor function and identified indications for utilizing VMI, which have not previously been determined.

### 4.1. Comparison of the Effects of P-VMI and S-VMI on Paralyzed Lateral Ankle Dorsiflexion Function

For dorsiflexion angular velocity, pre-intervention values did not differ between P-VMI and S-VMI, and post-intervention values were significantly higher for P-VMI. Although comparing changing rates between interventions showed no significant differences, only the values after the VMI intervention showed significant differences between P-VMI and S-VMI. The effect size of P-VMI was greater than that of S-VMI in terms of dorsiflexion angle and dorsiflexion angular velocity. In addition, SoA and SoO were higher with P-VMI than with S-VMI.

SoA is generated by the coincidence of motor intention and visual feedback and influences corticospinal tract excitability [26]. Minohara et al. [27] reported that adding a sense of effort enhanced SoA. Therefore, P-VMI, with its increased exercise intensity, may induce a greater sense of effort than S-VMI, resulting in a higher SoA and stronger activation of the tibialis anterior muscle, which may have a greater effect on ankle dorsiflexion. Mizuguchi et al. [25] reported that the excitability of corticospinal tracts depends on the magnitude of imagined muscle contraction. Because VMI induces the same motor imagery as the joint motion being observed, it is thought that the P-VMI images could have induced a strong contraction of the tibialis anterior muscle. This strong imagery may have a greater impact on the dorsiflexion function of the ankle joint on the paralyzed side by inducing greater SoA. Conversely, another reason SoO was greater in P-VMI than in S-VMI is that SoA and SoO are reported to interact, representing different experiences by exclusive brain regions but partially overlapping at the neural level [48]. Thus, SoO may have been significantly larger in P-VMI, such as SoA.

### 4.2. Relationship between the Degree of Improvement of VMI on the Paralyzed Side of the Ankle Joint Function and the SoA

Matsumiya et al. [49] used virtual reality in healthy subjects to investigate whether motor function is related to SoA or SoO. The results showed that SoA, but not SoO, correlated with motor function. VMI has been reported to activate frontoparietal networks [17]. The SoA has been reported to be important for activity in the parietal lobes and prefrontal cortex, which are involved in planning voluntary actions [50] and may overlap with the brain regions activated by VMI. Furthermore, Miyawaki et al. [51] evaluated motor function and SoA in hemiplegic patients over time and reported that SoA increased as motor function improved. Therefore, SoA may better reflect motor function, and the degree of improvement in ankle dorsiflexion function by VMI may be related to SoA.

### 4.3. Relationship between Cognitive Flexibility and Degree of Improvement in Ankle Joint Function by P-VMI and S-VMI

The degree of improvement in dorsiflexion angle and dorsiflexion angular velocity with P-VMI was negatively correlated with TMT-B (reflecting working memory). Previous studies have reported significant negative correlations between the degree of improvement in motor function with AOT and cognitive flexibility [29]. However, in this study, only the degree of improvement in dorsiflexion function with P-VMI but not with S-VMI was found to be associated with cognitive flexibility.

Gabbard et al. [52] examined the role of cognitive flexibility in the ability to translate motor imagery from videos into actual movement. Their results suggested that a higher task difficulty in the video required cognitive flexibility capacity and increased internalization of the model.

In this study, the image presented in P-VMI included resistance to elicit a sense of effort, while the image presented in S-VMI was that of a normal dorsiflexion movement, without resistance. P-VMI requires imagining a stronger contraction of the tibialis anterior muscle than S-VMI, suggesting that the amount of information that must be extracted from the video and retained is greater. Therefore, it is possible that a higher cognitive flexibility capacity is required for P-VMI and that the degree of improvement in ankle joint function with P-VMI is related to cognitive flexibility capacity.

### 4.4. Relationship between the Degree of Improvement in Ankle Joint Function and Physical Function with the Use of P-VMI and S-VMI

In the present study, the degree of improvement in dorsiflexion angular velocity with P-VMI correlated negatively with the degree of ankle plantar flexor muscle tone. Previous studies have reported that, in hemiplegic patients, effortful ankle dorsiflexion increases triceps muscle tone and the degree of joint contraction [53]. However, Flansbjer et al. [54] reported that progressive resistance training for hemiplegic patients with mild spasticity was effective in improving lower-extremity muscle strength and the subsequent degree of muscle tone. The mean CSS of participants in this study was 9.4 ± 2.1 points at baseline with P-VMI, which indicates a mild muscle tone. Therefore, even P-VMI, which elicits effortful imagery, showed a significant improvement in dorsiflexion angular velocity, given that the present study incorporated many cases with a low muscle tone.

The degree of improvement in dorsiflexion function with P-VMI and S-VMI was not correlated with the degree of lower extremity function or sensory impairment in the subjects. It is possible that the study did not include cases with severe lower extremity dysfunction and thus did not show a correlation due to the low variability in the severity of lower extremity function.

This study had several limitations. First, we did not measure the brain activity or corticospinal tract excitability. Second, the intervention in this study examined a single immediate effect, and it is unclear whether this effect persists. Further studies are needed to examine the long-term effects of this intervention in a larger number of cases. Third, this study examined the effect of a single joint in patients with hemiplegia. Future studies should examine the effects of P-VMI on movements, such as sit-to-stand and walking activities.

This study examined the immediate effects of VMI and confirmed the onset of P-VMI and S-VMI effects. The degree of ankle dorsiflexion function improvement with VMI was less than that of MCID and did not provide clinical benefit. However, although the degree of improvement in ankle dorsiflexion function is small, few previous studies have investigated the immediate effects of VMI, confirming the onset of the VMI effect. In addition, no previous studies have compared the two types of VMI immediate effect. This study may provide a basis for the application of VMI intervention methods. Future studies are needed to determine if long-term interventions can provide clinically beneficial changes in VMI.

## 5. Conclusions

P-VMI improved ankle function more than S-VMI, and the degree of improvement was related to the degree of SoA, cognitive flexibility capacity, and muscle tone during VMI. In particular, only the degree of improvement in dorsiflexion function with P-VMI was related to cognitive flexibility capacity as well as the degree of muscle tone. Based on these results, when adapting P-VMI to hemiplegic patients, it is necessary to consider their cognitive flexibility capacity and degree of muscle tone.

## Figures and Tables

**Table 1:** Post hoc power calculations for primary outcomes.

Outcomes	Power (1-β)	Effect Size
P-VMI		
Ankle dorsiflexion angle	0.99	0.96
Ankle dorsiflexion angular velocity	0.97	0.86
S-VMI		
Ankle dorsiflexion angle	0.84	0.67
Ankle dorsiflexion angular velocity	0.53	0.46

Abbreviations: P-VMI, Power-VMI; S-VMI, Standard-VMI; VMI, visual-motor illusion.

**Table 2:** Characteristics of the two groups.

Variable	Group A (*n* = 11)	Group B (*n* = 11)	*p* Value
Age (years)	60.3 (16.5)	64.5 (8.1)	0.459 ^a^
Gender (male/female)	8/3	5/6	0.193 ^c^
Time since stroke (days)	97.1 (28.5)	90.4 (29.9)	0.596 ^a^
Paralyzed side (right/left)	6/5	4/7	0.392 ^c^
Lower FMA (points)	20.3 (3.1)	21.2 (3.6)	0.533 ^a^
MAS	2.6 (0.7)	2.0 (1.1)	0.217 ^b^
MMSE (points)	27.6 (2.7)	28.6 (2.2)	0.353 ^a^

Notes: ^a^, independent *t*-test; ^b^, Mann–Whitney U test; ^c^, Chi-square test. Abbreviations: FMA, Fugl-Meyer assessment; MAS, Modified Ashworth Scale; MMSE, Mini-Mental State Examination.

**Table 3:** Results of ankle dorsiflexion function.

	P-VMI		S-VMI	
	Pre	Post	Pre	Post
Ankle dorsiflexion angle	15.7 (7.6)	18.5 (8.7) *	14.9 (8.5)	16.9 (7.9) *
Ankle dorsiflexion angular velocity	32.1 (22.6)	39.1 (26.7) *^†^	30.9 (22.5)	34.2 (24.1) *

Values indicate mean (standard deviation). There was a significant difference in ankle dorsiflexion angle between pre and post for each VMI. There was a significant difference in ankle dorsiflexion angular velocity between pre and post for each VMI, but P-VMI was significantly higher than S-VMI in post values. * Significant difference between pre and post. ^†^ Significant difference between P-VMI and S-VMI. Abbreviations: P-VMI, Power-VMI; S-VMI, Standard-VMI; VMI, visual-motor illusion.

**Table 4:** Results of correlation analysis between degree of improvement in ankle dorsiflexion function by VMI and various variables.

Degree of Improvement in Ankle Dorsiflexion Function	Various Variables	Correlation Coefficient	*p*-Value
**P-VMI**			
Dorsiflexion angle	Sense of ownership	0.248	0.266
	Sense of agency	**0.518**	**0.014**
	TMT-B	**−0.570**	**0.006**
	FMA	0.124	0.266
	SIAS-LE touch	0.071	0.754
	SIAS-LE position	0.049	0.828
	CSS	−0.179	0.426
Dorsiflexion angular velocity	Sense of ownership	0.019	0.857
	Sense of agency	**0.449**	**0.036**
	TMT-B	**−0.449**	**0.036**
	FMA	0.094	0.678
	SIAS-LE touch	0.029	0.898
	SIAS-LE position	0.009	0.970
	CSS	**−0.430**	**0.047**
**S-VMI**			
Dorsiflexion angle	Sense of ownership	0.360	0.100
	Sense of agency	**0.493**	**0.020**
	TMT-B	−0.257	0.248
	FMA	0.066	0.950
	SIAS-LE touch	0.178	0.428
	SIAS-LE position	0.186	0.406
	CSS	0.290	0.177
Dorsiflexion angular velocity	Sense of ownership	0.387	0.076
	Sense of agency	0.366	0.093
	TMT-B	−0.351	0.109
	FMA	−0.114	0.613
	SIAS-LE touch	0.071	0.754
	SIAS-LE position	0.081	0.720
	CSS	0.119	0.590

Abbreviations: P-VMI, Power-VMI; S-VMI, Standard-VMI; VMI, visual-motor illusion; TMT-B, Trail Making Test-B; FMA, Fugl-Meyer assessment; SIAS, stroke impairment assessment set; LE, lower extremity; CSS, Composite spasticity score; MMT, Manual muscle test; MMSE, Mini mental state examination.

## Data Availability

Participants in this study have not consented to the release of their data, so no supporting data is available.
